# Neutralizing and binding antibody dynamics following primary and booster COVID-19 vaccination among healthcare workers

**DOI:** 10.1186/s12879-025-10621-2

**Published:** 2025-02-14

**Authors:** Irmak Güzel, Gamze Öztürk, Özgür Appak, Derya Çağlayan, Ahmet F. Süner, Çağlar Irmak, Neslişah Türe, Elif Işik, Muammer Çelik, Gül Ergör, Alp Ergör, Yücel Demiral, Sema Alp Çavuş, Bulent Kilic, Arzu Sayiner

**Affiliations:** 1 Department of Medical Microbiology, Turkish Republic Ministry of Health, Nusaybin State Hospital, Nusaybin, Turkey; 2 Department of Medical Microbiology, Faculty of Medicine, Dokuz Eylül University, Izmir, Turkey; 3 Department of Public Health, Division of Epidemiology, Turkish Republic Ministry of Health, Diyarbakır Provincial Health Directorate, Diyarbakır, Turkey; 4 Çaycuma District Health Directorate, Zonguldak, Turkey; 5 Infectious Diseases and Clinical Microbiology Unit, Hakkari Yüksekova State Hospital, Hakkari, Turkey; 6 Department of Public Health, Faculty of Medicine, Dokuz Eylül University, Izmir, Turkey; 7 Department of Infectious Diseases and Clinical Microbiology, Faculty of Medicine, Dokuz Eylül University, Izmir, Turkey; 8 Department of Public Health, Division of Epidemiology, Faculty of Medicine, Dokuz Eylül University, Izmir, Turkey

**Keywords:** Antibodies, Neutralizing, sVNT, BNT162b2, CoronaVac, Healthcare workers, SARS-CoV-2

## Abstract

**Background:**

Vaccine-induced neutralizing antibodies (NAbs) are key for COVID-19 protective-immunity. As the efficacy of SARS-CoV-2 vaccines declines over time and variants of the virus continue to emerge, the need for booster doses of vaccine remains on the agenda. The aim of this study was to assess NAbs dynamics and its correlation with anti-RBD IgG levels during the nine-month follow-up period after primary-CoronaVac vaccination and booster vaccinations to evaluate vaccination strategies.

**Methods:**

This prospective longitudinal observational study followed 226 healthcare workers who received primary (two doses CoronaVac) and booster (CoronaVac or BNT162b2) immunization. Serum samples were collected at four different time points, two after primary vaccination and two after booster. Anti-RBD IgG antibody levels were assessed with the SARS CoV-2 IgG-II-QUANT kit (Abbott, USA) and neutralizing antibody levels were determined with the ACE2-RBD-Neutralization-Assay (Dia-Pro, Italy) using a surrogate virus neutralization method. Factors affecting antibody response were analyzed. Statistical analysis was performed with IBM-SPSS-22.0.

**Results:**

One month after the second dose of CoronaVac, 79.2% of participants had NAb, but this had decreased to 49.7% by the fourth month and was influenced by smoking, BMI and chronic diseases. Boosters, regardless of type, significantly raised NAb levels. Heterologous vaccination yielded higher NAb and anti-RBD IgG responses. Both single or double-BNT162b2 boosters resulted in similar NAb responses. There was a strong correlation between anti-RBD IgG and NAb levels following CoronaVac vaccination, leading to the identification of predictive IgG threshold for the presence of NAb. The type of booster influenced the correlation strength and threshold-value.

**Conclusions:**

NAbs levels decreased rapidly after primary CoronaVac vaccination. Boosters significantly increased levels while the heterologous vaccine combination induced a greater response. Anti-RBD IgG levels were able to predict the NAb response, however the correlation varied by the vaccine type, NAb response strength and the time since vaccination.

**Supplementary Information:**

The online version contains supplementary material available at 10.1186/s12879-025-10621-2.

## Introduction

The Coronavirus disease 2019 (COVID-19) pandemic, caused by SARS-CoV-2, has resulted in millions of deaths and placed unprecedented strain on global healthcare systems [1]. In response, a diverse array of vaccines has been developed to control the spread of the virus and reduce mortality rates. COVID-19 vaccines have been developed using both traditional approaches, such as inactivated viruses, and innovative methodologies, including recombinant protein subunits, viral vector platforms, and mRNA technologies. These advancements leverage detailed insights into SARS-CoV-2 structure and mutations, enabling the design of vaccines with enhanced efficacy and safety [2, 3]. Many countries, including Turkey, prioritized the vaccination of healthcare workers (HCWs) to manage the burden on healthcare systems. In Turkey, the vaccination of HCWs began on January 14, 2021, with an inactivated SARS-CoV-2 vaccine (CoronaVac, Sinovac Biotech, Beijing, China) administered in two doses 28 days apart. Starting from July 1, 2021, booster doses were offered, using either CoronaVac or the mRNA-based BNT162b2 vaccine (Comirnaty; Pfizer, New York, USA, and BioNTech, Germany) [4].

The accurate and rapid assessment of neutralizing antibody (NAb) responses is essential for evaluating immunity against SARS-CoV-2. Neutralizing antibodies, particularly those targeting the receptor-binding domain (RBD) of the viral spike protein, play a critical role in preventing infection by blocking the interaction between the virus and the host cell receptor, angiotensin-converting enzyme 2 (ACE2) [5, 6].While the plaque reduction neutralization test (PRNT) is considered the gold standard for NAb detection, its labor-intensive nature, reliance on biosafety level 3 facilities, and time-consuming process limit its applicability in routine or large-scale studies [7, 8].

To overcome these challenges, surrogate virus neutralization tests (sVNTs) have been developed, offering a cost-effective and rapid alternative that can be performed in biosafety level 2 laboratories. These tests measure the inhibition of the RBD-ACE2 interaction, providing a practical tool for assessing neutralizing activity in diverse settings [9–11]. Additionally, serological assays quantifying anti-RBD IgG levels have shown promise as proxies for NAb detection, with studies demonstrating strong correlations between anti-RBD IgG titers and neutralizing activity [12–14].

However, the emergence of SARS-CoV-2 variants, particularly those with mutations in the spike protein, highlights the need for the ongoing evaluation of diagnostic tools. Variants such as Omicron, characterized by significant antigenic shifts, may reduce the accuracy of tests based on ancestral RBD antigens [15]. In resource-limited settings, it is particularly valuable to investigate protective immunity in high-risk groups using simpler and more cost-effective methods.

Despite numerous studies evaluating antibody responses following COVID-19 vaccination, there is limited data from Türkiye specifically addressing healthcare workers—a high-risk group with significant exposure to the virus—or examining the dynamics of antibody responses after heterologous and homologous booster regimens in real-world settings.

This prospective longitudinal study evaluates the NAb response in healthcare workers using the sVNT method following primary vaccination and homologous or heterologous booster doses. It also monitors changes in NAb levels during follow-up and examines the correlation between NAb and anti-RBD IgG levels to estimate protective immunity based on anti-RBD IgG results. By offering detailed insights into the dynamics of neutralizing and binding antibodies, the study aims to enhance the understanding of immune responses in this critical population.

## Methods

### Study design and population selection

This study is a continuation of a prospective longitudinal research conducted at Dokuz Eylül University Hospital in Turkey, with the initial results previously published [16]. The present study monitored antibody responses in healthcare workers (HCWs) who received two doses of the CoronaVac vaccine, followed by either homologous or heterologous booster doses, over a nine-month period from March 4 to December 31, 2021.

Systematic sampling was used to select 560 HCWs for participation, as detailed in reference [16]. Out of these, 226 HCWs provided written informed consent and consistently followed the study protocol by contributing blood samples at specified intervals. Data on sociodemographic characteristics and factors that may affect vaccine response were collected through a questionnaire during the first blood draw (An additional file provides further details on the questionnaire [see Additional file 1]). The factors assessed included the presence of chronic diseases, immunosuppressive treatment, smoking (nonsmokers, smokers, former smokers), and body mass index (BMI) (< 24.9 kg/m^2^: lean-normal, 25–29.9 kg/m^2^: overweight, > 30.00 kg/m^2^: obese).

Participants who received the booster dose were planned to be categorized into two groups based on the time interval between booster vaccination and blood sample collection to evaluate the effect of timing on antibody responses. This categorization included intervals of 7–45 days (short interval), and 46–67 days (long interval). To maintain the consistency of booster timing and ensure the reliability of antibody response assessments, participants who provided blood samples outside the specified timeframe of 7 to 67 days after the first booster dose were excluded from the analysis.

Participants who were symptomatic or in contact with persons with confirmed SARS-CoV-2 infection were tested using reverse transcription-polymerase chain reaction (RT-qPCR) on nasopharyngeal swab samples. Participants diagnosed with COVID-19 during the study period were excluded to avoid confounding effects of natural infection on antibody response analyses.

**Fig. 1:**
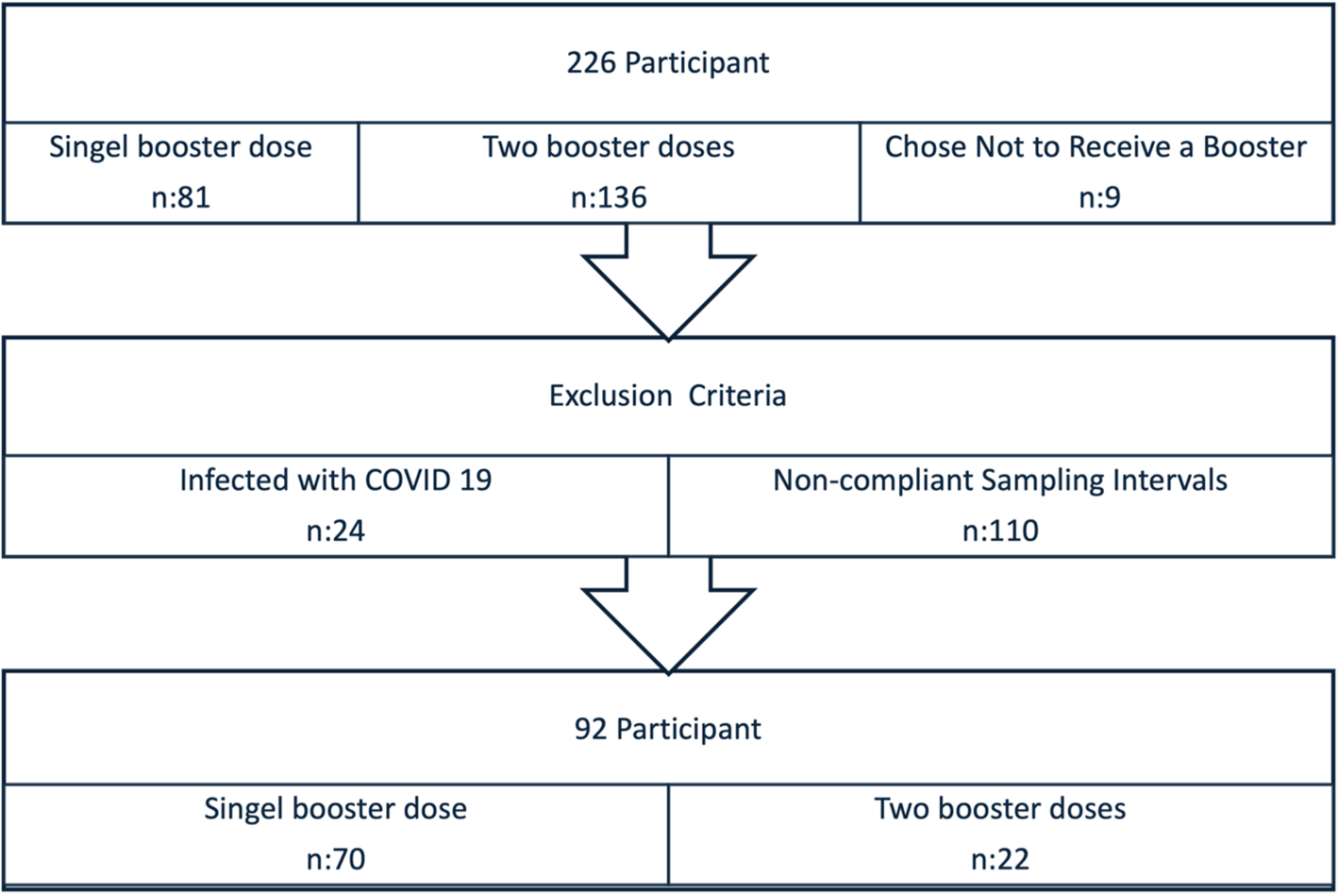
Flow diagram illustrating the enrollment, exclusions, and final grouping of participants based on booster dose choices

The exclusions were based on predefined criteria to ensure a homogeneous study population and minimize potential sources of bias. By adhering to these criteria, the study aimed to provide robust and interpretable results regarding the dynamics of neutralizing and binding antibody responses following primary and booster COVID-19 vaccination. Figure 1 illustrates the flow of participants, including exclusions and grouping based on booster dose administration.

### Blood collection

**Fig. 2:**
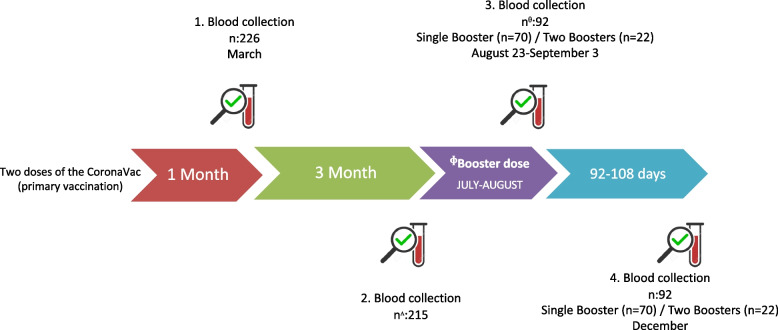
Postvaccination blood sample collection timeline in healthcare workers: CoronaVac primary and booster doses

The timeline of blood sample collection is illustrated in Fig. 2. Blood samples were collected at four time points during the study:

1. The first sample was obtained one month after the primary two-dose CoronaVac vaccination.
2. The second sample was collected four months after the primary vaccination.
3. HCWs were offered the choice of a booster dose (CoronaVac or BNT162b2) between July and August 2021. The third sample was collected after, between August 23 and September 3, 2021.
4. The fourth sample was collected in December 2021, approximately 92–108 days after the third blood draw.

### Determination of antibody levels

#### Detection of anti-RBD IgG antibodies

The SARS-CoV-2 IgG II QUANT (Abbott, Chicago, IL, USA) test used is a chemiluminescent immunoassay*** (***CLIA). It quantifies IgG-type antibodies against the RBD of the viral S-protein in serum samples using the Abbott Architect platform (Abbott, IL, USA), with a range of 50- 40.000 AU/mL, where ≥ 50 AU/mL serves as the positivity threshold.

#### Detection of ACE2-RBD neutralizing antibodies

The ACE2-RBD neutralization test (Dia-Pro, Italy) used is an ELISA-based semiquantitative sVNT that assesses the inhibition activity of RBD-ACE2 binding induced by antibodies against ancestral SARS-CoV-2 in serum samples. The neutralization value (%) of each sample was calculated according to the manufacturer’s recommended formula (100- (optical density (OD)_450nm_Sample / meanOD_450nm_Negative Control) × 100). Values ​​ ≥ 20% were considered positive and classified into three categories: weak (20–29%), good (30–59%), and excellent (≥ 60%).

### Outcome and covariates

The antibody levels were used as the outcome variables of the study. Age, gender, smoking status, BMI, presence of chronic diseases, and immunosuppressive-modulatory treatment status were considered as covariates.

### Statistical analysis

Descriptive statistics were presented as counts and percentages for categorical variables, and as mean ± standard deviation (SD) or median (interquartile ranges (IQRs)) for numerical variables, depending on the normal distribution. The normal distribution of continuous variables was tested using the Kolmogorov–Smirnov or Shapiro–Wilk tests. As the antibody levels did not follow a normal distribution, the Mann–Whitney U test was used to compare independent groups, the Kruskal–Wallis test for comparisons involving more than two groups, and the Wilcoxon test for comparisons within dependent groups. Predictor IgG levels for NAb values ≥ 20% and ≥ 60% were assessed using receiver operating characteristics (ROC) curve analysis. The cutoff point was determined by calculating the Youden index. Numerical variables that did not meet the normal distribution criteria were analyzed by the Spearman correlation test. The correlation coefficient (r) threshold values were considered as follows: ( +) positive, (-) negative direction and 0.75–1.00 indicate a very strong correlation, 0.50–0.74 indicate a strong correlation, 0.25–0.49 indicate a moderate correlation, and 0.24 and below indicates a weak correlation. Analyses were performed using SPSS 22.0 (IBM Corporation, Armonk, New York, United States), and *p*-value < 0.05 was considered to indicate statistical significance.

## Results

We included 226 HCWs in the study, with a mean age of 41.1 years (SD = 10.2; range: 23–65). Table 1 presents the participants' age groups, gender, smoking status, BMI, presence of chronic diseases, and immunosuppressive treatment status. Seventy-one of the participants (31.4%) had at least one chronic disease, and 5 (2.2%) were receiving immunosuppressive treatment. Fourteen participants had more than one multiple chronic disease.

**Table 1:** Characteristics of study group and neutralizing antibody levels at first and fourth months

**Characteristic**	**Number**	**1**^**st**^** month**	**4**^**th**^** month**
	**n (%)**	**NAbs (%) median IQR**	**NAbs (%) median IQR**
**Gender**			
Female	162 (75.3)	59.37 (28.82–72.41)	21.65 (2.44–42.95)
Male	53 (24.7)	56.31 (22.25–75.75)	17.67 (0–46.86)
*p* value		0.916	0.587
**Age (years)**			
20–35	65 (30.2)	62.69 (35.20–79.76)	24.42 (3.15–49.40)
36–50	102 (47.4)	59.37 (19.53–71.86)	19.23 (0.74–38.95)
51–65	48 (22.3)	53.70 (24.14–70.99)	13.74 (0–44.06)
*p* value		0.238	0.469
**Smoking**			
Nonsmokers	127 (59.1)	59.08 (34.25–75.79)	24.03 (5.50–46.26)
Smokers	49 (22.8)	59.62 (14.64–70.87)	10.91 (0–35.80)
Former smokers	39 (18.1)	46.51 (8.62–71.78)	10.13 (0–42.46)
*p* value		0.084	**0.03**
**Body mass index**^a^			
Lean-normal	103 (48.4)	63.37 (38.39–77.51)	25.14 (4.94–46.04)
(18.5–24.9)			
Overweight	81 (37.7)	50.34 (16.60–69.08)	14 (0–36.56)
(25- 29.9)			
Obese	29 (13.5)	51.07 (13.22–72.12)	21.19 (0.94–40.02)
(> 30)			
*p* value		**0.011**	0.53
**Chronic disease**			
Yes^c^	71 (33)	48.56 (14.88–69.60)	13.22 (0–37.12)
No	144 (67)	60.85 (35.09–75.38)	23.67 (3.56–47.45)
*p* value		**0.02**	**0.021**
**Immun-suppressive/modulatory therapy**			
Yes	5 (2.3)	6.02 (0–62.25)	10.13 (4.54–41)
No	210 (97.7)	60.02 (28.79–73.90)	19.82 (1.10–43.25)
*p* value		0.095	0.806
**Total study group**	215^b^	57.7 (24.4–73)	19.01 (0.08–42.9)
Positivity (% n/N)		79.2	49.7
*p* value		**< 0.001**	

*IQR* Interquartile range, *Nabs* Neutralizing antibodies

^a^Body mass index data were not available for two people

^b^Eleven infected individuals were excluded from the study

^c^Chronic diseases: Hypertension (*n* = 21), asthma-allergy (*n* = 9), rheumatologic disease (*n* = 8), diabetes mellitus (*n* = 4), cardiovascular disease (*n* = 5), cancer (active or in remission) (*n* = 3),

chronic lung disease (*n* = 4), chronic neurologic disease (*n* = 1), chronic liver disease (*n* = 1) and asplenia (*n* = 1)

### NAb and anti-RBD IgG response following primary vaccination with CoronaVac

We detected NAbs in 79.2% (179/226) of the participants one month after the second dose of CoronaVac, but this declined to 49.7% (107/215) by the fourth month. At months 1 and 4 median NAb values were 57.7% (IQR: 24.4–73.0) and 19.01% (IQR: 0.08–42.9), respectively (*p* < 0.001). The NAb results by group according to sociodemographic characteristics and factors that may affect the vaccine response are presented in Table 1.

**Fig. 3:**
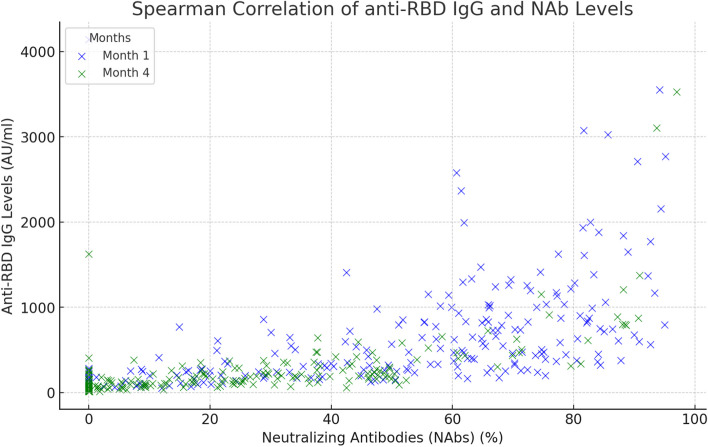
Correlation of anti-RBD IgG and NAb levels at month 1 vs. month 4. Spearman correlation of anti-RBD IgG and NAb levels at both the 1st and 4th months. A strong positive correlation was observed in both months (*r* = 0.702, *p* < 0.001 in the 1st month and *r* = 0.720, *p* < 0.001 in the 4th month)

There were no significant differences in NAb values at the first or fourth month according to age, gender, or immunosuppressive treatment status; however, smoking, BMI, and chronic disease status influenced NAb levels. Individuals in the lean-normal BMI group presented higher NAb levels at the 1st month (*p* = 0.01), whereas nonsmokers presented higher NAb levels at the 4th month (*p* = 0.03). Participants with chronic diseases had significantly lower NAb values at both evaluations (*p* = 0.02; *p* = 0.02) (Table 1). A strong positive correlation was observed between anti-RBD IgG and NAb levels in participants who tested positive for both antibodies at the 1st and 4th month assessments after two doses of CoronaVac (Fig. 3). However, 104 participants had positive anti-RBD IgG (≥ 50 AU/mL) but negative NAb values (< 20%) in at least one of the two assessments.

### Anti-RBD IgG cutoff values to predict NAb response

**Fig. 4:**
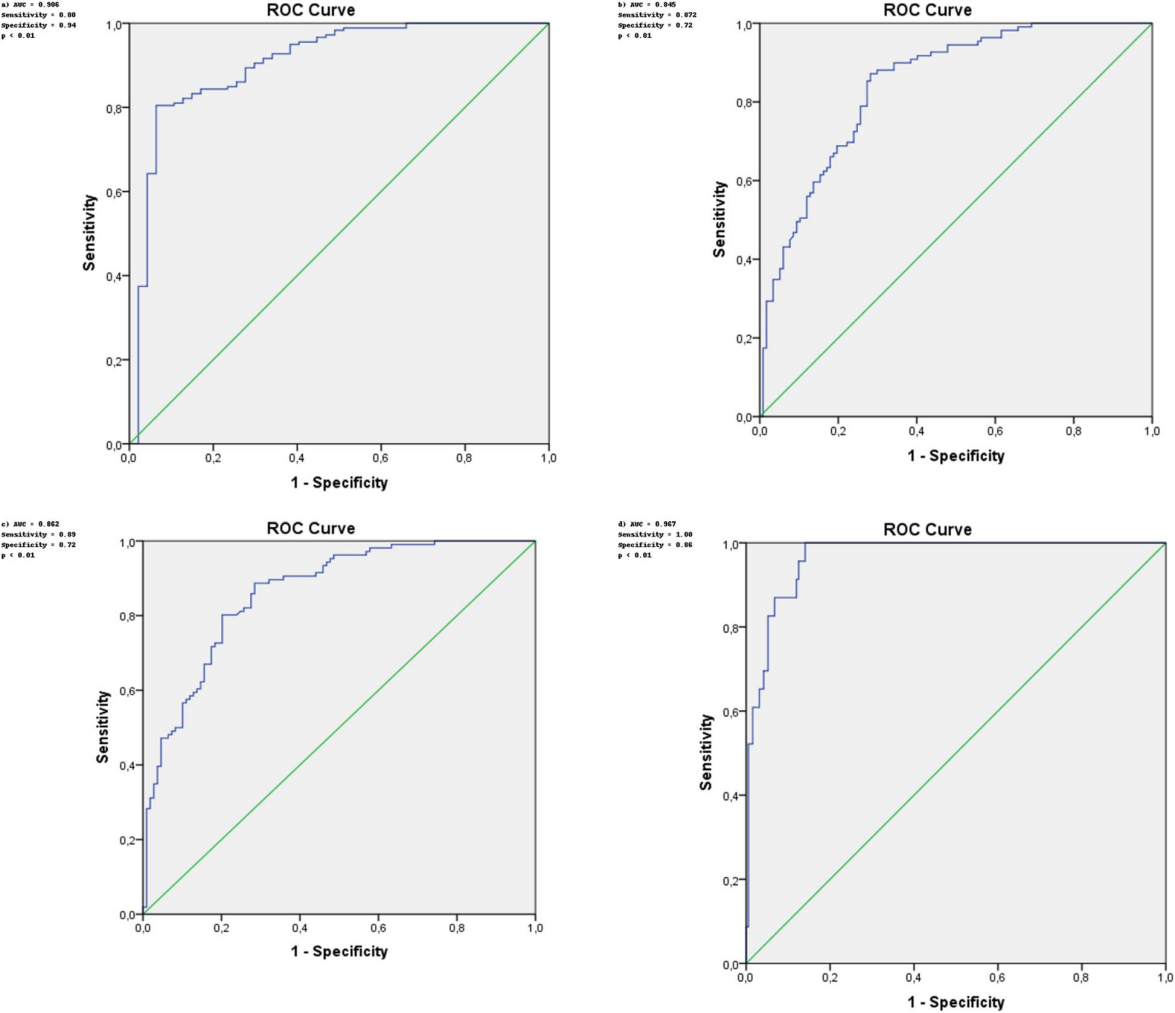
Receiver operating characteristic (ROC) curves for anti-RBD IgG levels predicting neutralizing antibody (NAb) positivity at months 1 and 4. 1st month ROC analysis for predicting NAb ≥ 20% (a) and NAb ≥ 60% (b), 4th month ROC analysis for predicting NAb ≥ 20% (c) and NAb ≥ 60% (d),

The ROC and Youden index were used to calculate the relationships between the levels of anti-RBD IgG and two different neutralization thresholds (≥ 20% and ≥ 60%) after two doses of CoronaVac (Fig. 4). The cutoff values for anti-RBD IgG to predict NAb positivity have shown variability depending on the assessment time and the selected NAb value. Anti-RBD IgG levels that predicted NAb positivity (≥ 20%) at month 1 and month 4 were 277.25 AU/mL and 131.45 AU/mL, respectively. The IgG levels predictive of excellent NAb levels (≥ 60%) at these time points were calculated to be 357.8 AU/mL and 302.85 AU/mL, respectively.

### Anti-RBD IgG and NAb levels after the booster vaccination

#### Participants with a single booster dose

Among the 70 participants, 84% (*n* = 59) chose the BNT162b2 vaccine as their booster, while 16% (*n* = 11) preferred CoronaVac. There was a minimum of 7 days, and a maximum of 67 days between the administration of the booster dose and the collection of the 3rd blood sample. Of the participants included in the analysis, 12 individuals (16%) were classified in the "short interval" group (7–45 days), and 58 individuals (84%) were classified in the "long interval" group (46–67 days). Table 2 shows NAb levels between these two groups were similar (*p* > 0.05).

**Table 2:** Comparison of NAb Levels Following Booster Dosing at Different Time Intervals

Neutralizing antibody levels following boooster dose		
Time between booster dose and blood collection	NAb (%)	
	3rd blood sample	4th blood sample
Short interval: 7–45 days (n: 12)	97.2	96.7
Long interval: 46–67 days (n: 58)	96.3	95.5
p	0.119	0.269

*NAb* Neutralizing antibody

When the participants are analyzed by vaccine type, significant differences in immune responses are observed. Below, the data are presented separately for participants receiving the BNT162b2 booster (a) and the CoronaVac booster (b).

a BNT162b2 booster group

Participants who received the BNT162b2 booster (*n* = 59) exhibited a significant decline in anti-RBD IgG levels between the 3rd and 4th assessments (from 18.750.9 AU/mL at the 3rd assessment to 5.118.1 AU/mL at the 4th assessment, *p* < 0.001; Table 3). Despite this decline, NAb levels remained consistently high over time, with values of 96.59% at the 3rd and 96.32% at the 4th assessment (*p* > 0.05; Table 3). This stability in NAb levels was observed even in the presence of a wide range of anti-RBD IgG levels among participants (166.4–40.000 AU/mL). Correlation analysis further supports this observation, suggesting that the NAb response is not strongly dependent on changes in anti-RBD IgG levels (rho = + 0.3 at the 3rd assessment; rho = + 0.5 at the 4th assessment). The variability in anti-RBD IgG levels, coupled with the consistently high NAb levels across participants, made it challenging to determine specific anti-RBD IgG cutoff values for NAb levels using ROC analysis. In addition, sociodemographics, chronic medical conditions, immunosuppressive treatment, smoking status and BMI were analyzed in participants who received the BNT162b2 booster. None of these factors had a significant effect on vaccine response (*p* > 0.05).

**Table 3:** Evaluation of Anti-RBD IgG and Neutralizing Antibody Responses After BNT162b2 and CoronaVac Vaccinations

**Levels of anti-RBD IgG and NAb after a single booster dose**					
**Measurement**	**BNT162b2 (*n* = 59)**	**CoronaVac (*n* = 11)**	***p*-value (BNT162b2 vs CoronaVac)**	***p*-value (3rd vs 4th, BNT162b2)**	***p*-value (3rd vs 4th, CoronaVac)**
3rd anti-RBD IgG (AU/mL)	18750.9	1598.4	*p* < 0.001	*p* < 0.001	*p* < 0.001
4th anti-RBD IgG (AU/mL)	5118.1	323.4	*p* < 0.001	-	-
3rd NAb (%)	96.59	93.71	*p* < 0.01	*p* > 0.05	< 0.001
4th NAb (%)	96.32	77.65	*p* < 0.001	-	-

*NAb* Neutralizing antibody

b CoronaVac booster group

Participants who received the CoronaVac booster (*n* = 11) had significantly lower antibody levels compared to the BNT162b2 group. At the 3rd assessment, their anti-RBD IgG levels were 1598.4 AU/mL, which further decreased to 323.4 AU/mL by the 4th assessment (*p* < 0.001, Table 3). Similarly, their NAb levels declined significantly, from 93.71% at the 3rd to 77.65% at the 4th assessment (*p* < 0.001). Notably, two participants in the CoronaVac group had undetectable NAb levels at the 4th assessment.

#### Participants with two booster doses

All 22 participants who received two booster doses, selected the BNT162b2 mRNA vaccine. As shown in Table 4, anti-RBD IgG levels were significantly higher in participants receiving two doses than in those receiving a single dose of BNT162b2 (*p* < 0.05). NAb levels were > 96% in both groups.

**Table 4:** Neutralizing antibody and Anti-RBD IgG levels in the fourth sample after single/double doses of BNT162b2

**Antibody levels at 4th sample after single/double BNT162b2 doses measurement**		
n	Anti-RBD IgG (AU/mL)^*^	NAb (%)
Single dose BNT162b2 (*n*: 59)	5118.1	96.32
Double dose BNT162b2 (*n*: 22)	10208.7	96.92

*NAb* Neutralizing antibody

^*^Significant at *p* < 0.05

#### Participants who did not receive a booster dose

During the study period, nine participants out of 226 did not receive any booster after the primary vaccination with two doses of the CoronaVac. In this group, two participants had detectable anti-RBD IgG levels; however, no detectable NAb was present in any of the four assessments in the study. In one person, the levels of anti-RBD IgG and NAb detected at baseline gradually decreased and no NAb was found in the last two assessments. In another participant, the NAb values remained above the excellent level (≥ 60%) in all four blood samples evaluated. The remaining five participants became infected with COVID-19 during the follow-up period.

#### Participants infected with COVID-19

During the nine-month period, 24 participants were infected. The mean age of the infected participants was 37.8 years (SD = 9.1; range: 23–54), and 71% were female. The mean BMI of the infected participants was 24.79 (SD = 3.8; range: 20–34). Among them, 29% were smokers, and 17% were former smokers. Five participants had at least one chronic illness, and one was receiving immunosuppressive treatment.

Among the 24 infected, 11 were detected in the first 3 months of the study and the other 13 were detected in the second period when the supplementary doses were administered. Among these participants, 59% (14/24) tested positive for SARS-CoV-2 RNA before any booster dose, 33% (8/24) after a single dose of BNT162b2 and 8% (2/24) after two doses of BNT162b2. Those infected after the booster received no further vaccines during the study period. The symptoms observed in infected participants were of mild to moderate severity, with no cases requiring hospitalization.

## Discussion

Neutralizing antibodies against SARS-CoV-2 are critical indicators of immune protection and vaccine efficacy. In this study, we evaluated NAbs among HCWs over nine months following primary CoronaVac vaccination and booster doses, using anti-RBD IgG and the sVNT. Although the plaque reduction neutralization test (PRNT) is widely regarded as the gold standard for measuring NAbs, it is time-intensive and requires enhanced biosafety measures due to the use of live virus. We used the sVNT, a rapid, easy-to-use assay, to evaluate the inhibition of RBD-ACE2 binding activity. The sVNT is widely recognized as a reliable tool for assessing NAb responses in real-world settings due to its high specificity (> 95%) and sensitivity (> 90%) across studies [17, 18].

Our results showed that one month after receiving the second dose of CoronaVac, nearly 80% of those enrolled had detectable NAb levels. However, this response declined rapidly, with only about 50% of participants maintaining detectable levels by the fourth month. There was also a significant decrease in median NAb levels. These results are in line with previous studies that have reported similar rapid decline in antibody levels following primary vaccination with inactivated virus vaccines [19, 20]. The humoral immune response following vaccination can also be influenced by host factors such as age, gender, and chronic diseases [21–23]. In this prospective longitudinal study of HCWs, age and gender did not significantly affect NAb response, contrary to some previous reports [24, 25]. However, non-smokers and those with a lean-normal body mass index (BMI) had higher NAb levels, whereas participants with chronic diseases had lower antibody levels. Consistent with our findings, Indrati et al. reported decreased antibody responses in smokers, those with comorbidities and obesity [26]. They suggested that pro-inflammatory cytokine profiles and B cell dysfunctions may impair adaptive immune responses, while smoking was specifically linked to compromised memory cell formation and increased antibody clearance, contributing to the diminished NAb levels in these groups.

Our findings further demonstrated strong positive correlations between anti-RBD IgG and NAb levels in participants who received CoronaVac for primary vaccination. Using ROC analysis, anti-RBD IgG cutoff values were identified to predict NAb levels of ≥ 20% and ≥ 60%. At one-month post-vaccination, these cutoff values were 277.25 AU/mL and 357.8 AU/mL, respectively, decreasing to 131.45 AU/mL and 302.85 AU/mL by the fourth month.

These decreases are consistent with previous reports suggesting that the IgG thresholds required to achieve similar levels of neutralization decrease over time due to antibody affinity maturation, allowing lower antibody concentrations to maintain comparable neutralizing effects [27, 28]. Consistent with our findings, Lee et al. demonstrated that the Abbott SARS-CoV-2 IgG II Quant kit, used in our study, exhibited the strongest correlation with PRNT titers among multiple assays, particularly in predicting neutralization capacity related to the RBD region [29]. Similarly, Cin et al. also observed a strong correlation between anti-S IgG levels and NAb titers in individuals with a history of COVID-19 vaccination and/or SARS-CoV-2 infection [30]. Despite the strong correlations observed in our study, 104 participants had at least one instance where anti-RBD IgG values were positive (≥ 50 AU/mL), but NAb values were considerably low (< 20%). Similar discrepancies between CLIA-based IgG measurements and NAb levels have also been reported by Indrati et al. [26]. They attributed discrepancies to interfering factors, including rheumatoid factor, heterophile antibodies, complement, and cross-reactive antigens. Cross-reactivity in CLIA methods, potentially caused by human metapneumovirus (HMPV), common cold coronaviruses, influenza virus, or rhinovirus, was also suggested as a contributing factor. These factors may be relevant to discrepancies observed in our results.

Of the 92 participants who received booster doses, the majority opted for a heterologous booster. The booster administration effectively raised NAb levels above 60% and, restored neutralizing immunity regardless of the type of booster vaccine used. Similarly, participants who received two booster doses of BNT162b2 achieved high NAb levels comparable to those who received a single booster dose, with no significant differences observed between the two groups. In contrast to those who opted for homologous booster regimens, NAb levels in both groups receiving BNT162b2 boosters remained consistently stable above 96% throughout the 3-month follow-up period. Participants who received heterologous boosters demonstrated consistently higher NAb and anti-RBD IgG levels compared to those who received homologous boosters. Notably, NAb levels fell below the detectable threshold in two participants who received a homologous booster, while no such cases were observed in those who received a heterologous booster throughout the evaluation period. These results, similar to findings reported in other studies [31–33], underscore the advantages of heterologous regimens in sustaining neutralizing immunity.

Anti-RBD IgG levels increased after booster administration but declined during the follow-up. Participants receiving heterologous boosters maintained elevated NAb levels, suggesting additional mechanisms contributing to sustained neutralization. Subjects receiving BNT162b2 boosters had consistent NAb levels regardless of host factors such as age, sex, or smoking status. In contrast to primary vaccination, where certain host factors influenced the NAb response, booster doses maintained consistently high NAb levels in all groups. Heterologous boosters enhance RBD-specific memory B cell and S1-specific T cell responses, contributing to stronger immunity and cross-protection against SARS-CoV-2 variants [34, 35]. These cellular immune responses, along with mechanisms such as affinity maturation or the presence of long-lived plasma cells, may explain the sustained NAb levels observed in heterologous booster recipients. In addition, the robust NAb responses elicited by heterologous regimens may result from broader cross-binding and specific activation of memory B and T cells, enhancing their potential to maintain protective immunity in diverse settings [36–38].

In our study, the observed weakening of the correlation between anti-RBD IgG and NAb levels may be attributed to the heterologous regimen. While our study group received two doses of inactivated vaccine followed by one or two mRNA boosters, the study by Grunau et al. focused on immune responses after two doses of a primary mRNA vaccine. Their results showed moderate positive correlations between SARS-CoV-2 antibody levels and RBD-ACE2 binding inhibition, which differ from our study’s dynamics. Notably, Grunau et al. found stronger correlations 2–3 months after vaccination than immediately after [39]. This highlights the complexity of immune responses in mixed vaccination strategies and underscores the need for further research to clarify these dynamics.

During the nine-month period, 24 participants (10.6%) were excluded from our study due to infection. Of these, approximately 60% tested positive for SARS-CoV-2 RNA before receiving any booster dose after the primary vaccination. Although the SARS-CoV-2 variant was not identified in these cases, the predominant variant in Turkey from March and April 2021 was alpha, followed by delta. A case–control study conducted during a similar timeframe, reported that individuals who received two doses of CoronaVac were more likely to become infected than those who received a combination of booster doses with CoronaVac and BNT162b2 [40]. No significant differences in antibody responses were observed between those who became infected and those who remained uninfected, regardless of the vaccination regimen. These findings are consistent with our observations and highlight the ongoing challenge of defining NAb levels that can reliably predict protection from infection. With the ongoing impact of the Omicron variant, studies have demonstrated a reduction in immune responses against emerging variants that partially evade neutralization [41, 42]. However, the need and timing of additional booster doses remain unclear. Although our study focused on antibodies against the ancestral strain, other studies have shown that a third dose of an mRNA vaccine following inactivated vaccines can induce NAb titers against the Omicron variant, even if levels against the ancestral strain are reduced [32, 43]. In addition, consistent with our findings, studies evaluating mRNA boosters have shown that NAb levels remain stable over time, suggesting that booster dose intervals could be extended [44–46].

The findings from our study highlight the fundamental role of booster doses in maintaining sustained neutralizing immunity. While anti-RBD IgG levels serve as a practical surrogate marker for NAbs and offer viable solutions for monitoring immunity, booster doses may complicate their reliability as a predictive measure. In addition, the observed decline in anti-RBD IgG levels over time underscores the potential need for complementary NAb assays to provide a more accurate assessment of immune responses.

There are several limitations of this study. First, some participants were excluded due to noncompliance with the specified timing for booster vaccine administration and subsequent blood sampling. This exclusion reduced the overall sample size, particularly for certain subgroups, and limited the ability to perform comprehensive analyses. Second, although the plaque reduction neutralization assay considered the gold standard for NAb detection, it has not been used due to its time-consuming nature, requirement for specialized laboratory conditions, and challenges associated with its application in a large population. Third, this study assessed only RBD-specific antibodies in relation to NAb levels, which may not fully capture the contribution of antibodies targeting other epitope regions to neutralization. Fourth, the NAbs evaluated were specific to the ancestral strains included in the vaccines studied. Since viral mutations can alter antibody neutralization, ongoing evaluation of immunity against emerging variants is essential. Finally, while the focus of the study on HCWs, a high-risk group, provides valuable insights, the limited distribution of age and potential comorbidities is a limitation. This also creates challenges in generalizing the findings to a broader population.

## Conclusion

In conclusion, this study provides insight into the effects of homologous and heterologous (BNT162b2) booster vaccines on anti-RBD IgG and NAb responses following primary CoronaVac vaccination. Our findings can guide vaccination strategies and inform assessments of long-term effects.

## Supplementary Information

Supplementary Material 1.

## Data Availability

The data supporting the findings of this study are available within the article. All other data are not publicly available owing to concerns regarding participant anonymity and confidentiality. However, these data are available upon request from the corresponding author, subject to ethical guidelines and approval.Clinical trial number: not applicable. author, subject to ethical guidelines and approval. Clinical trial number: not applicable.

## Abbreviations

NAbs: Neutralizing antibodies
COVID-19: Coronavirus disease 2019
HCWs: Healthcare workers
S: Spike
RBD: Receptor-binding domain
PRNT: Plaque reduction neutralization test
sVNT: Surrogate virus neutralization test
BMI: Body mass index
RT-qPCR: Reverse transcription-polymerase chain reaction
CLIA: Chemiluminescent *immunoassay*
OD: Optical density
SD: Standard deviation
IQR: Interquartile range
ROC: Receiver operating characteristics
HMPV: Human metapneumovirus
