# Supplementary material for: Neutralizing and binding antibody dynamics following primary and booster COVID-19 vaccination among healthcare workers
Source: BMC Infect Dis. 2025 Feb 14;25:218. doi: 10.1186/s12879-025-10621-2 (PMC11827177; doi:10.1186/s12879-025-10621-2)
Supplement: Supplementary file 1 — Supplementary Material 1. [file 12879_2025_10621_MOESM1_ESM.pdf]

DOKUZ EYLUL UNIVERSITY HOSPITAL: NINE-MONTH FOLLOW-UP STUDY OF ANTIBODY RESPONSE TO COVID-19 VACCINE IN HEALTHCARE WORKERS

SURVEY FORM

Date: 04.03.2021
Version: 1.0

Survey No:
Interview Date:
Interviewer’s Name:

Information:

This study, conducted by Dokuz Eylul University Hospital, is titled “Cohort Study of Neutralizing and Binding Antibody Dynamics After Primary and Booster Vaccination in Healthcare Workers Using a Surrogate Virus Neutralization Test”
This study aims to:
- Determine the seroconversion rate in individuals who have received two doses of the CoronaVac vaccine without prior COVID-19 infection.
- Evaluate the neutralizing antibody responses after primary and booster vaccinations and determine their correlation with anti-RBD IgG antibodies detected by simple CLIA tests, along with identifying cutoff values.
- Monitor changes in detectable antibody levels to determine the duration of the vaccine's protective effect.

The data collected from participants will be used solely for scientific purposes and will not be shared with third parties.

A. SOCIODEMOGRAPHIC CHARACTERISTICS

1. Full Name:
2. Barcode No:
3. Age:
4. Gender:
   1. Female
   2. Male
5. Height (cm):

6. Weight (kg):
7. Occupation:
- Physician
- Nurse
- Other healthcare worker: (Please specify)

B. GENERAL HEALTH AND RISK FACTORS ASSESSMENT

1. Do you have any of the following conditions? If so, please mark with an X:
- Hypertension
- Chronic heart disease
- Diabetes
- Chronic lung disease
- Chronic kidney disease
- Chronic liver disease
- Rheumatic disease
- Cancer
- Organ transplant
- Bone marrow transplant
- Immunoglobulin/complement deficiency
- Asplenia (Spleen removal or loss of function)

2. If you have any other conditions not listed above, please specify:
3. Are you taking any immunosuppressive medications (e.g., for rheumatic diseases)? If so, please specify:
- Yes, I take _________
- No
4. Have you received any immunosuppressive treatment (e.g., chemotherapy, radiotherapy) in the past year?
- Yes
- No
5. Are you currently using corticosteroids? If yes, please provide the name and dosage:
- Yes. Drug name: _____ Dosage: _____ mg
- No

6. Do you smoke? Please select the appropriate option:
- Yes, I currently smoke
- No, I have never smoked
- I quit smoking (Please specify when: _______)

7. Were you pregnant at the time of your first or second vaccine dose?
- Yes
- No

C. VACCINATIONS

1. When did you receive your second dose of the COVID-19 vaccine?
2. Did you receive a flu vaccine between September 2020 and February 2021?
   - Yes, Date: ______/______ (Month/Year)
   - No
3. If you received the flu vaccine, was the interval between the flu vaccine and the COVID-19 vaccine less than 15 days?
   - Yes
   - No
4. Did you receive a pneumococcal vaccine in the past 6 months?
   - Yes, Date: ______/______ (Month/Year)
   - No
5. If you received the pneumococcal vaccine, was the interval between the pneumococcal vaccine and the COVID-19 vaccine less than 15 days?
   - Yes
   - No
6. Did you receive any other vaccines within a month prior to your COVID-19 vaccines?
   - Yes, I received __________ vaccine(s)
   - No

This section will be completed by the interviewer:

1. Has the participant been involved in the study for more than 1 year?
   - Yes
   - No
2. In case of an unforeseen situation (e.g., change of workplace, retirement), was the participant informed about continuing the study from outside the institution?
   - Yes
   - No
